# Short-term amblyopic eye deprivation boosts the binocular intermodulation in anisometropic amblyopia

**DOI:** 10.3389/fnins.2026.1780127

**Published:** 2026-04-01

**Authors:** Siyuan Deng, Fang Li, Wenbin Huang

**Affiliations:** Hainan Eye Hospital and Key Laboratory of Ophthalmology, Zhongshan Ophthalmic Center, Sun Yat-sen University, Haikou, Hainan, China

**Keywords:** amblyopia, eye dominance plasticity, reverse deprivation, short-term monocular deprivation, steady-state visual evoked potentials

## Abstract

**Purpose:**

We tested whether short-term monocular deprivation (STMD) of the amblyopic eye enhances binocular intermodulation steady-state visual evoked potentials (SSVEPs), reflecting reduced suppression from the fellow eye.

**Methods:**

Fifteen anisometropic amblyopes (15.6 ± 5.8 years) underwent 2-h STMD of their fellow eye on the first day, followed by a similar session on their amblyopic eye on the second day. We recorded SSVEPs from 21 occipital electrodes pre/post STMD during binocular rivalry (6/7.5 Hz flicker).

**Results:**

Similar to the previous study, the monocular SSVEP amplitude of the deprived eye increased after STMD, whereas that of the non-deprived eye remained unchanged. Critically, STMD of the amblyopic eye significantly increased f*1*+f*2* intermodulation amplitude (*t* = 2.42, pFDR = 0.030), while STMD of the fellow eye showed no such effect (*p* > 0.05). Baseline f*1*+f*2* amplitude was strongly correlated with the magnitude of SSVEP change following STMD (*r* = 0.789, *p* < 0.001).

**Conclusion:**

STMD of the amblyopic eye, but not the fellow eye, significantly enhanced binocular SSVEP intermodulation. Baseline f1+f2 amplitude predicted the magnitude of plasticity, suggesting its potential as an electrophysiological biomarker for reverse deprivation therapy.

## 1 Introduction

Amblyopia is the most common condition causing visual impairment in children worldwide (Birch, 2013). The prevalence ranges from 2% to 5% globally and it is the most common cause of monocular blindness in clinical practice (Sturm, 1994). Amblyopia results from early visual development deficits caused by strabismus, refractive error, anisometropia, or visual deprivation, leading to monocular or binocular visual impairment. Beyond its effects on vision, amblyopia significantly impacts patients’ quality of life and mental health, placing a substantial social and economic burden on society. Deprivation of the fellow eye was first utilized to treat amblyopia by de Buffon (2005), and today conventional deprivation is widely used as the first line of amblyopia therapy (Levi, 2020; Rajavi et al., 2019). In an effort to boost treatment success rates, practitioners began using reverse deprivation, pleoptics, and red filters starting around 1945 (Malik et al., 1975). Clinical research dating back to the 1960’s have attempted to treat amblyopic patients using reverse deprivation in an effort to enhance paracentral fixation (Andree, 1966; Cibis and Windsor, 1967; Malik et al., 1969). In the 1970’s, standard deprivation was once more the go-to treatment for all patients under the age of seven because reverse deprivation therapies had not proven to be as effective as initially anticipated. However, children often show poor compliance with fellow-eye patching, and the treatment may be associated with psychosocial impacts (Holmes et al., 2003; Webber et al., 2008). At least 50% of patients have experienced improvement in their amblyopic eye’s visual acuity with depriving the fellow eye. However, regaining binocular vision is rarely successful. Recent research has revealed that amblyopes with 20/20 vision after deprivation therapy nevertheless demonstrated unbalanced binocular vision (Gong et al., 2023). Since some believe that the loss of binocular function is the underlying deficiency (with loss of monocular visual acuity as a result), greater focus is currently being placed on creating more effective binocular therapies. In fact, restoring binocular function might have more functional advantages as well as being more important from an etiological standpoint (Hess, 2022).

Visual plasticity is the capacity of the visual system to be changed by visual experience. In patients with amblyopia, corresponding homeostatic plasticity was discovered in both fellow eyes and amblyopic eyes. Short-term monocular deprivation (STMD) affects the binocular balance by making the non-deprived eye’s contribution weaker and the previously deprived eye’s contribution greater. It was discovered that amblyopic observers experienced longer-lasting changes in visual plasticity than their normal counterparts (Gong et al., 2023; Lunghi et al., 2016; Zhou et al., 2015). According to these findings, it is necessary to deprive the amblyopic eye, not the fellow eye, in order to improve binocular vision. Recent research on adolescent and adult amblyopes have proven the binocular effectiveness of this novel strategy. Understanding the neural process of reverse deprivation is necessary in order to properly implement this new treatment plan in the clinical setting. The accepted theory currently holds that there are two possible mechanisms of reverse deprivation for amblyopia: (1) depriving the fellow eye to improve access to the amblyopic eye, and (2) depriving the fellow eye to lessen its inhibitory effect on the amblyopic eye, which leads to increased visual acuity.

Electroencephalograph (EEG) also provides a way to reveal cortical responses after visual stimulation. EEG possess good temporal resolution and lightweight mobility features. With the improvement in the performance of computer and the optimization of EEG signal acquisition equipment, the number of studies on extracting visual task-related results from EEG signals of amblyopic patients has increased gradually (Lunghi et al., 2016; Zhou et al., 2015; Baker et al., 2015; Bankó et al., 2013; Körtvélyes et al., 2012). A common feature of EEG studies of binocular rivalry function in amblyopia is the application of flashing optotypes with different frequency tagging to differentiate the signals received by the two eyes and to generate steady-state VEPs (SSVEPs) of different frequencies in the corresponding cortex (Brown and Norcia, 1997; Tang and Norcia, 1995). By analyzing the intensity of the EEG signals at these two different frequencies in real time, it is possible to obtain a quantitative EEG index of binocular visual signals in the visual center in real time.

In this study, we were particularly interested in the neurological basis of STMD with a view to its potential application in the treatment of amblyopia in order to lessen the well-known suppressive imbalance. We used the same binocular rivalry paradigm as in our earlier studies to measure the SSVEP amplitudes in amblyopes in order to evaluate the monocular and binocular interactions before and after STMD. We chose steady-state VEPs (SSVEPs) over transient VEPs for the following reasons: (1) SSVEPs allow simultaneous frequency-tagging of the two eyes’ inputs (6 vs. 7.5 Hz), enabling separate tracking of monocular and binocular cortical responses; (2) the intermodulation components (f1+f2) specifically index binocular interaction, which was our primary outcome; (3) SSVEPs offer a higher signal-to-noise ratio and faster data acquisition compared to transient VEPs, which is advantageous in clinical populations; (4) this paradigm has been extensively validated in amblyopia research (Gu et al., 2020; Zhou et al., 2015). While transient VEPs are valuable for assessing visual acuity and contrast sensitivity (Yap et al., 2020), SSVEPs are better suited for quantifying interocular suppression and binocular integration. The binocular rivalry SSVEP paradigm used in this study yields stable topographic maps and is well-suited for quantifying interocular suppression and binocular integration in amblyopia (Baitch and Levi, 1988; Zhang et al., 2011). Here, we hypothesized that STMD of the amblyopic eye (reverse deprivation) would enhance binocular intermodulation in the visual cortex, and that baseline SSVEP measures would predict this plasticity. This hypothesis aligns with emerging evidence on experience-dependent plasticity in amblyopia.

## 2 Materials and methods

### 2.1 Subjects

Fifteen adolescent and adult (15.6 ± 5.8 years; four female) with anisometropic amblyopia participated in this study. Clinical data for these participants are presented in Table 1. All participants had been stably wearing their optimal optical correction for at least 3 months prior to the study to ensure stable and adapted refractive status, in line with standard practice in amblyopia research. A written informed consent was obtained from each of them before the start of the test. This study complied with the Declaration of Helsinki and was approved by the Institutional Review Boards of Hainan Eye Hospital. The methods were carried out in accordance with the approved guidelines.

**TABLE 1:** Clinical information on patients with anisometropic amblyopia.

Subject	Age (years)	Sex	OD refractive error (SE, D)	OS refractive error (SE, D)	BCVA fellow eye (logMAR)	BCVA amblyopic eye (logMAR)	Stereoacuity (arcsec)
1	25	F	+0.875	+2.50	−0.08	0.1	40
2	18	M	+3.25	0.00	−0.14	0.18	60
3	22	M	+1.75	+3.75	−0.18	0.4	/
4	8	M	+0.75	+3.25	−0.08	0.1	100
5	12	M	0.00	+4.75	−0.18	0.6	/
6	22	M	+1.25	+2.63	−0.08	0.22	/
7	12	M	+1.75	+3.75	−0.08	1	/
8	8	M	+4.50	+0.88	−0.08	0.22	200
9	24	M	+0.50	+5.75	−0.08	0.1	400
10	12	F	+3.75	0.00	−0.08	0.7	/
11	15	M	−0.25	+3.38	0	0.22	/
12	12	M	−2.25	+1.25	−0.08	0.1	40
13	9	F	0.00	−4.50	0	0.22	400
14	17	F	−3.00	−0.50	0	1.3	/
15	18	M	+9.25	+3.25	0.22	0.7	/

/, mean; non-measurable.

### 2.2 Apparatus

All stimuli were generated and controlled by a PC running Matlab (MathWorks, Natick, MA) with PsychTool Box 3.0.9 extensions (Brainard, 1997; Pelli, 1997). Binocular rivalry stimuli were presented on a 27-inch LCD monitor (ASUS) using an active shutter stereo-goggle (NVIDIA 3D Vision 2) at a mean luminance of 150 cd/m^2^. The monitor was gamma-calibrated at a refresh rate of 120 Hz to ensure a 60 Hz presentation in each eye. A chinrest was used to minimize the subjects’ head movements.

The EEG signals were amplified and digitized using a SynAmps 2 64-channel Amplifier with the 64-channel Quick-Cap in accordance with the international 10–20 system (Compumedics, United States), which allows fast and simple electrode placement. Signals were recorded from 21 posterior electrodes with a focus on covering the occipital scalp region, and the impedance of each electrode was kept below 10 kΩ. Horizontal and vertical electrooculograms (HEOG and VEOG) were also recorded to monitor eye movements. A reference electrode was placed between Cz and CPz. The data were sampled at 1,000 Hz and filtered with a 0.05–100 Hz bandpass filter.

### 2.3 Design

**FIGURE 1:**
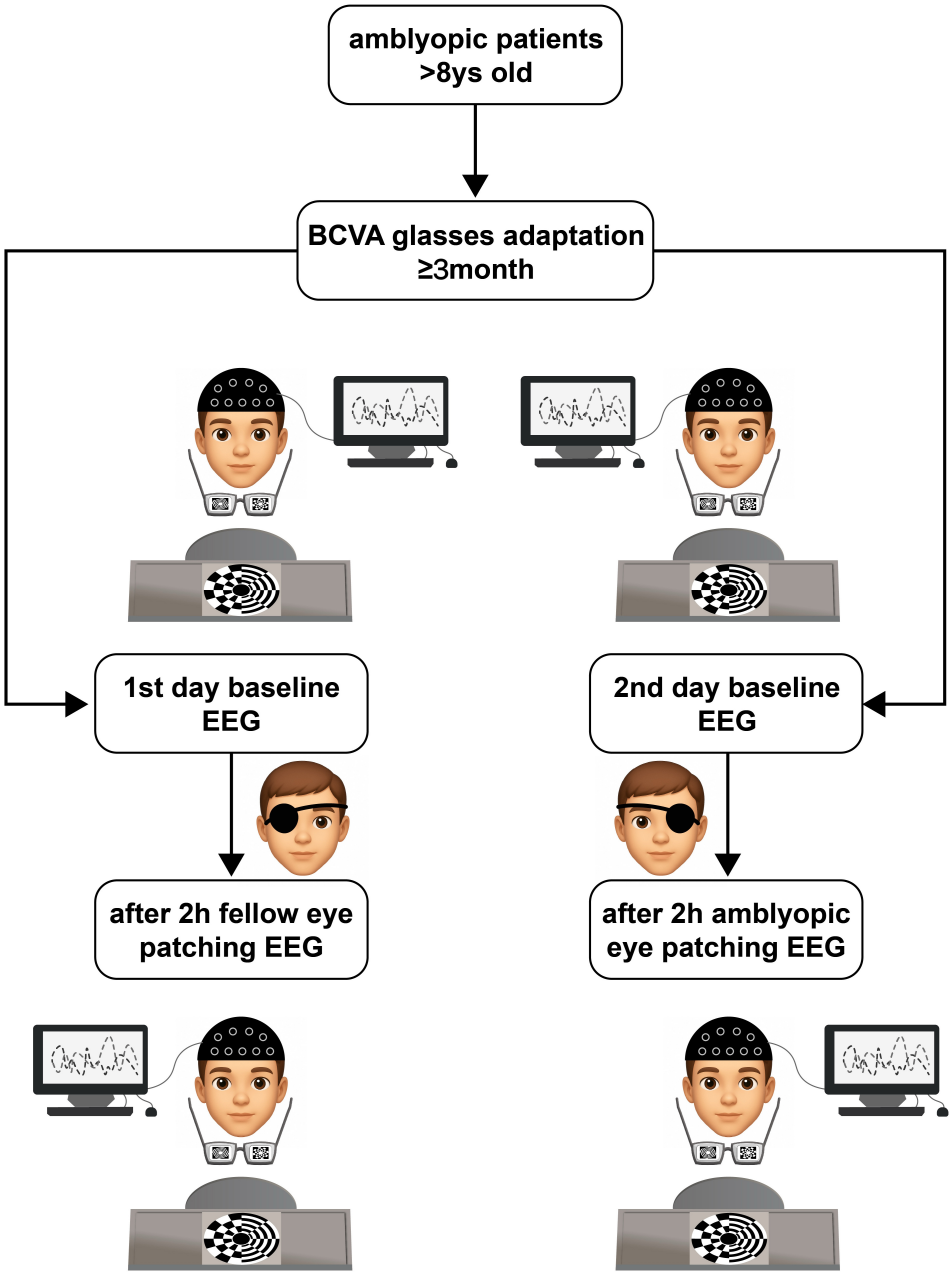
Experimental procedure. Participants underwent short-term monocular deprivation on the fellow eye for 2 h on 1^st^ day and short-term monocular deprivation of the amblyopic eye for 2 h on the subsequent day. Steady-state visual evoked potentials (SSVEPs) measurements were recorded both before and after each short-term monocular deprivation (STMD) session while the subjects viewed flickering binocular rivalry stimuli.

The experiment consisted of a 2-day consecutive procedure: (1) a first-day baseline SSVEP recording (230 s), a STMD of the fellow eye stage (2 h) with SSVEP recording (230 s; started immediately after the removal of the deprivation); (2) and a second-day baseline SSVEP recording (230 s), a STMD of the amblyopic eye stage (2 h) with SSVEP recording (230 s; started immediately after the removal of the deprivation). An opaque eye-patch was placed in front of the fellow eye during the first-day STMD of the fellow eye stage and the amblyopic eye during the second-day STMD of the amblyopic eye stage, respectively. The subjects were allowed to use a computer, read a book, or go for a walk while they were under STMD. In order to ensure that the electrode placements recorded before and after STMD were equal, the electrode positions were indicated before the experiment began (Figure 1). The two sessions were separated by a 24-h interval, which substantially exceeds the documented ∼2-h recovery time of STMD-induced plasticity (Lunghi et al., 2016; Zhou et al., 2015), thus serving as an adequate washout period. Baseline SSVEP amplitudes did not differ significantly between Day 1 and Day 2 (all *p* > 0.3), confirming no residual carry-over effects.

### 2.4 Stimuli

**FIGURE 2:**
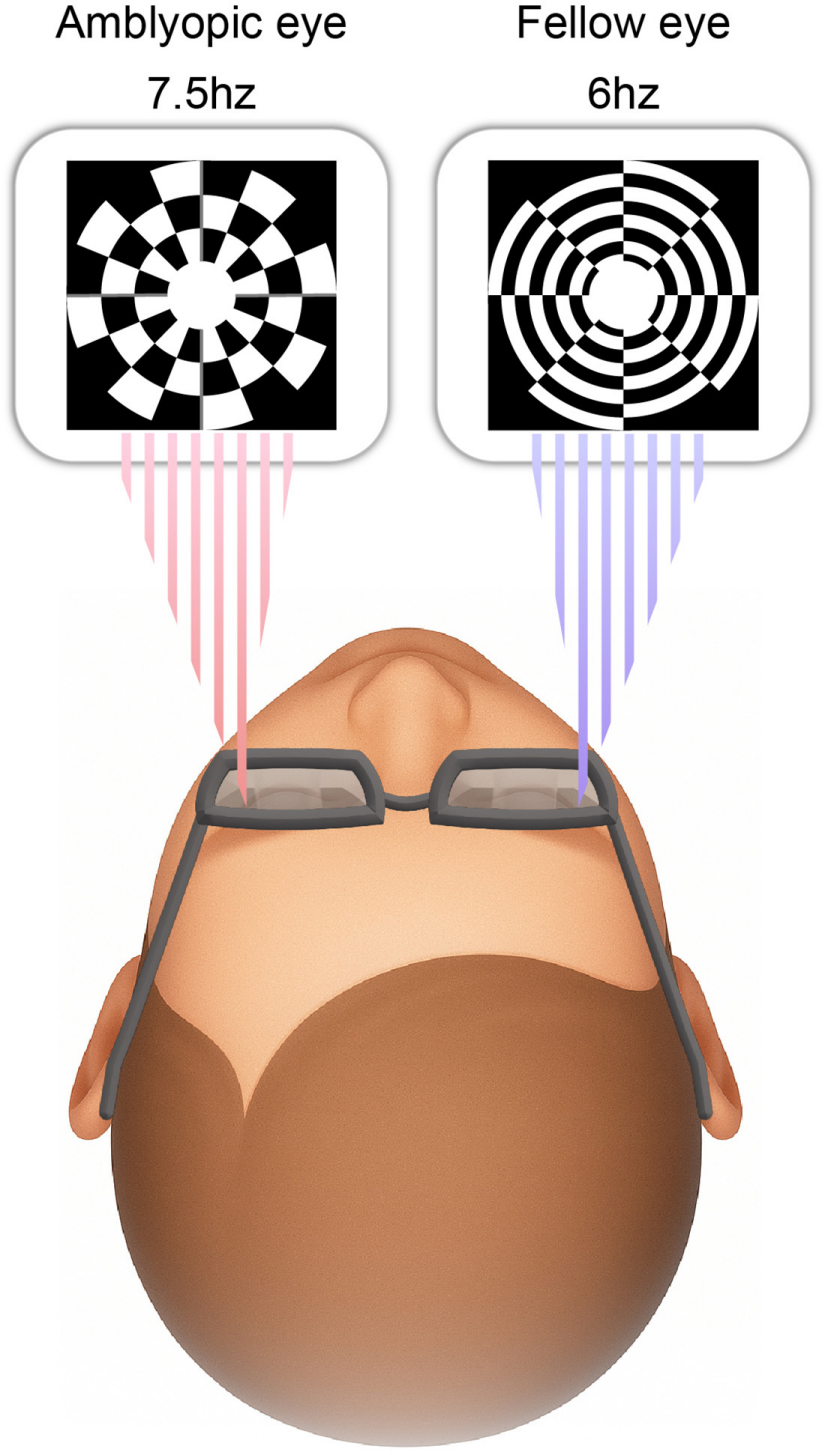
Binocular rivalry stimuli Binocular stimuli were simultaneously presented to each eye. Dichoptic circular checkerboard patterns were presented to the two eyes via shutter goggles. The pattern presented to one eye reversed contrast at 6 Hz, and the pattern presented to the other eye reversed contrast at 7.5 Hz.

A pair of incompatible circular checkerboard patterns adopted from a previous SSVEP binocular rivalry study was presented simultaneously to each eye through the goggles, with an annular window with a 10° visual angle (Zhang et al., 2011). The two patterns reversed their contrast at 6 and 7.5 Hz, respectively. Subjects viewed the display in a dark room at a distance of 1.0 m. Successive frames were seen by only one eye with no perceptible flicker at the high alternation rate. Subjects fixated on a central dark mark that remained visible throughout the experiment and actively monitored the parafoveal rivalrous stimuli. Each trial lasted 30 s, and each subject completed six trials with 10 s of rest between them (Figure 2).

### 2.5 Procedure for EEG recording

The subjects were seated in a shielded room. By stimulating the two eyes using stimuli flickering at two different frequencies, f_1_ and f_2_, we were able to tag the activities of monocular neurons according to EEG signals at the fundamental frequencies and their harmonics, m*f_1_ and n*f_2_, where m and n are integers. The activities of binocular neurons, which combine inputs from the two eyes and possess binocular non-linearities, such as rectification, squaring, and/or divisive normalization, were tagged by EEG signals at the non-linear intermodulation frequencies m*f_1_ ± n*f_2_ (Baitch and Levi, 1988; Regan and Regan, 1988; Zhang et al., 2011). Signals were recorded from 21 posterior electrodes with a focus on covering the occipital scalp region, and the impedance of each electrode was kept below 10 kΩ. Horizontal and vertical electrooculograms (HEOG and VEOG) were also recorded to monitor eye movements. A reference electrode was placed between Cz and CPz. The data were sampled at 1,000 Hz and filtered with a 0.05–100 Hz bandpass filter.

### 2.6 Statistics

Paired *t*-tests were conducted to compare pre- and post-STMD SSVEP amplitudes for the key frequency components (f*1*, f*2*, f*1*+f*2*) (Gu et al., 2020; Zhou et al., 2015). To control for multiple comparisons, we applied the Benjamini-Hochberg False Discovery Rate (FDR) procedure separately for the fellow-eye and amblyopic-eye deprivation sessions. FDR-corrected *p*-values are denoted as pFDR. Statistical significance was set at α = 0.05. Pearson correlation was used to assess the relationship between baseline f*1*+f*2* amplitude and Δf*1*+f*2*.

## 3 Results

### 3.1 Monocular EEG responses

Following 2 h of monocular deprivation, SSVEP amplitude at the fundamental frequency corresponding to the deprived eye increased significantly in both conditions.

**FIGURE 3:**
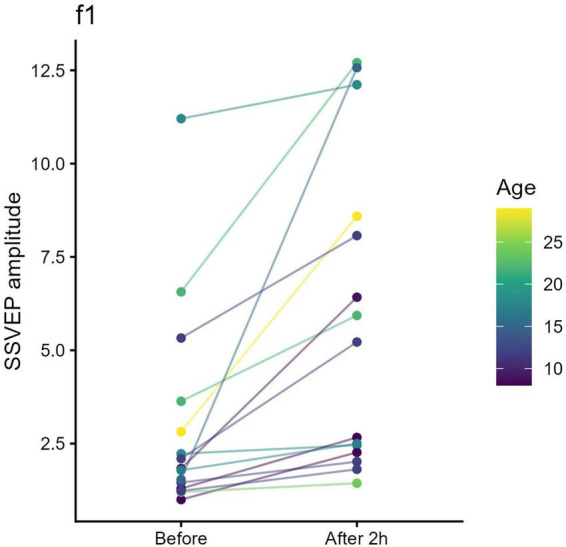
Monocular steady-state visual evoked potential (SSVEP) amplitude before and after short-term monocular deprivation (STMD) of the fellow eye (f1 = 6 Hz). Each colored line represents an individual participant. After 2 h of STMD of the fellow eye, SSVEP amplitude at the deprived eye’s tagging frequency (6 Hz) increased.

For STMD of the fellow eye (Day 1), amplitude at the deprived eye’s tagging frequency (f*1* = 6 Hz) increased from baseline (*t* = 3.57, pFDR = 0.031) (Figure 3), while no significant change was observed for the non-deprived amblyopic eye (f*2* = 7.5 Hz; *t* = 0.11, pFDR = 0.91).

**FIGURE 4:**
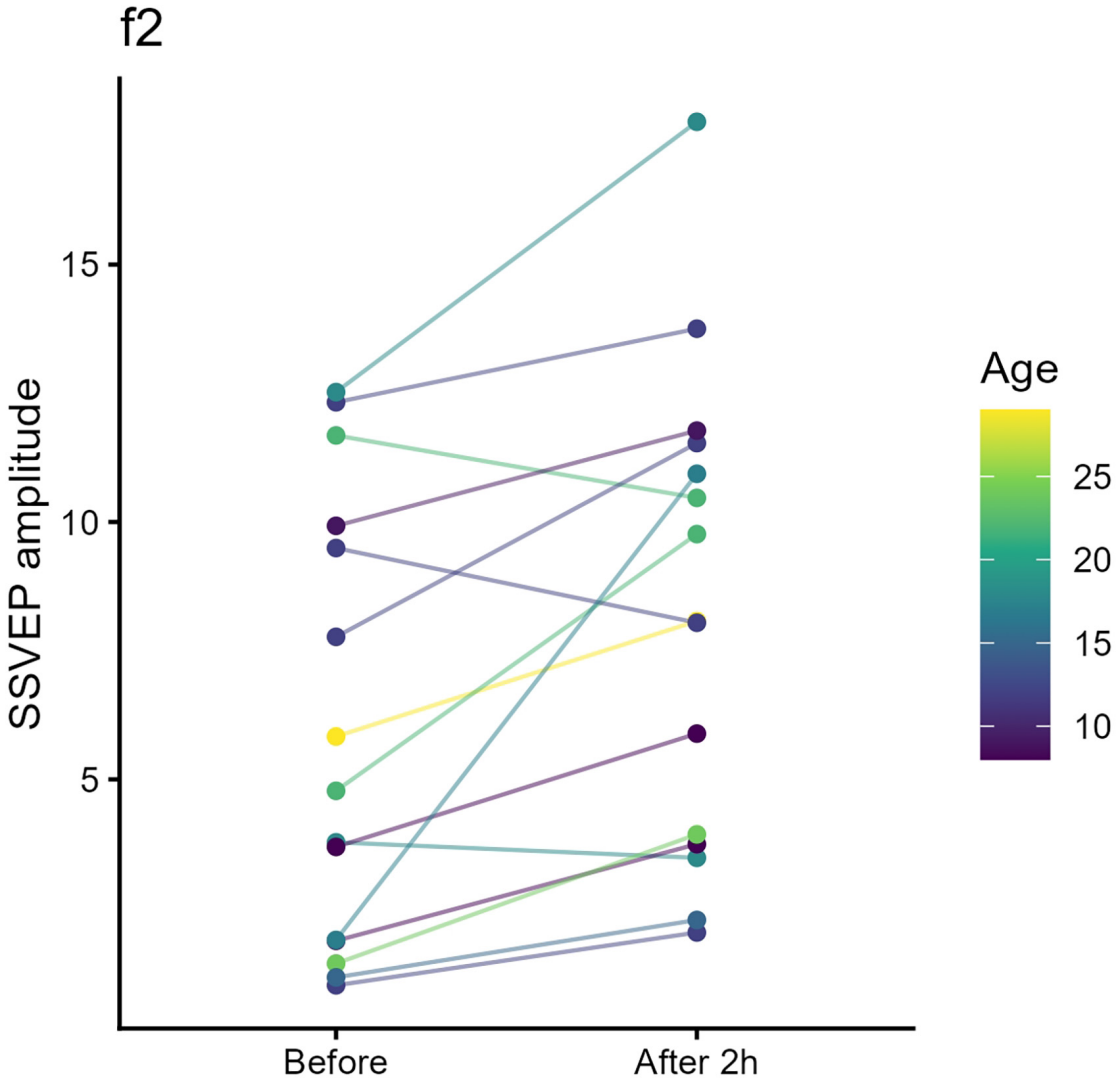
Monocular steady-state visual evoked potential (SSVEP) amplitude before and after short-term monocular deprivation (STMD) of the amblyopic eye (f2 = 7.5 Hz). Each colored line represents an individual participant. After 2 h of STMD of the amblyopic eye, SSVEP amplitude at the deprived eye’s tagging frequency (7.5 Hz) increased.

For STMD of the amblyopic eye (Day 2), amplitude at the deprived eye’s tagging frequency (f*2* = 7.5 Hz) increased significantly (*t* = 3.30, pFDR = 0.005) (Figure 4), with no significant change in the non-deprived fellow eye (f*1* = 6 Hz; *t* = 0.22, pFDR = 0.83).

These monocular effects replicate previous findings (Zhou et al., 2015; Lunghi et al., 2015) and confirm that our STMD manipulation successfully induced ocular dominance plasticity in both deprivation conditions.

### 3.2 Binocular SSVEP responses – intermodulation components

We next examined whether STMD modulated binocular interaction, as indexed by intermodulation SSVEP amplitudes (f*1*+f*2*).

**FIGURE 5:**
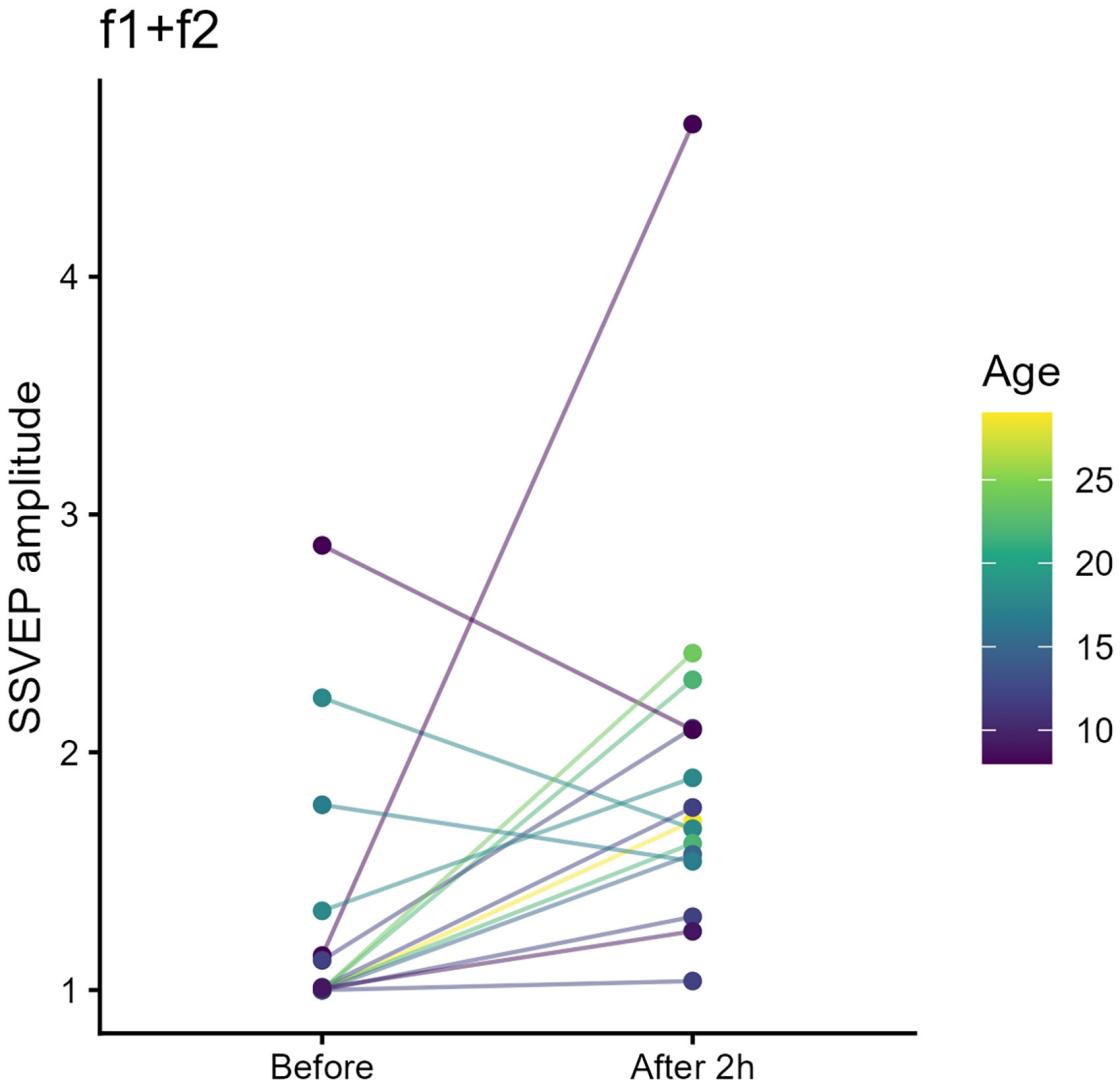
Binocular intermodulation steady-state visual evoked potentials (SSVEP) amplitude (f1+f2) before and after short-term monocular deprivation (STMD) of the amblyopic eye. Each colored line represents an individual participant. After 2 h of STMD of the amblyopic eye, f*1*+f*2* intermodulation amplitude increased, indicating reduced interocular suppression and enhanced binocular interaction.

Following STMD of the amblyopic eye, the f*1*+f*2* intermodulation component showed a significant amplitude increase (*t* = 2.42, pFDR = 0.030) (Figure 5).

**FIGURE 6:**
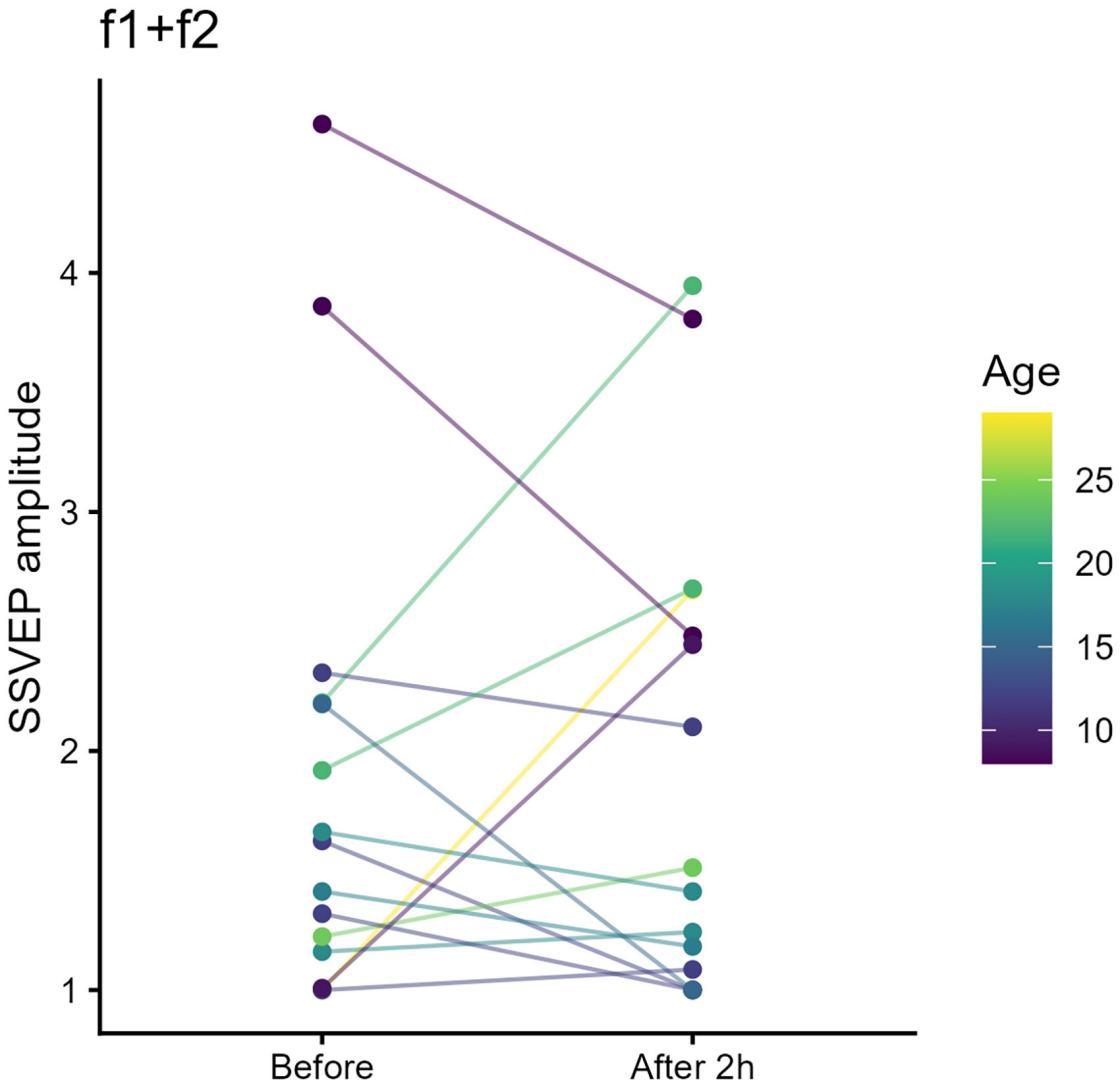
Binocular intermodulation steady-state visual evoked potentials (SSVEP) amplitude (f*1*+f*2*) before and after short-term monocular deprivation (STMD) of the fellow eye. Each colored line represents an individual participant. No systematic change in f*1*+f*2* intermodulation amplitude was observed following fellow-eye STMD, suggesting limited modulation of binocular interaction under this condition.

In contrast, following STMD of the fellow eye, no significant change was found at f*1*+f*2* (*t* = 0.28, pFDR = 0.79) (Figure 6).

These results demonstrate that only STMD of the amblyopic eye—not the fellow eye—enhances binocular cortical responses in anisometropic amblyopia.

**FIGURE 7:**
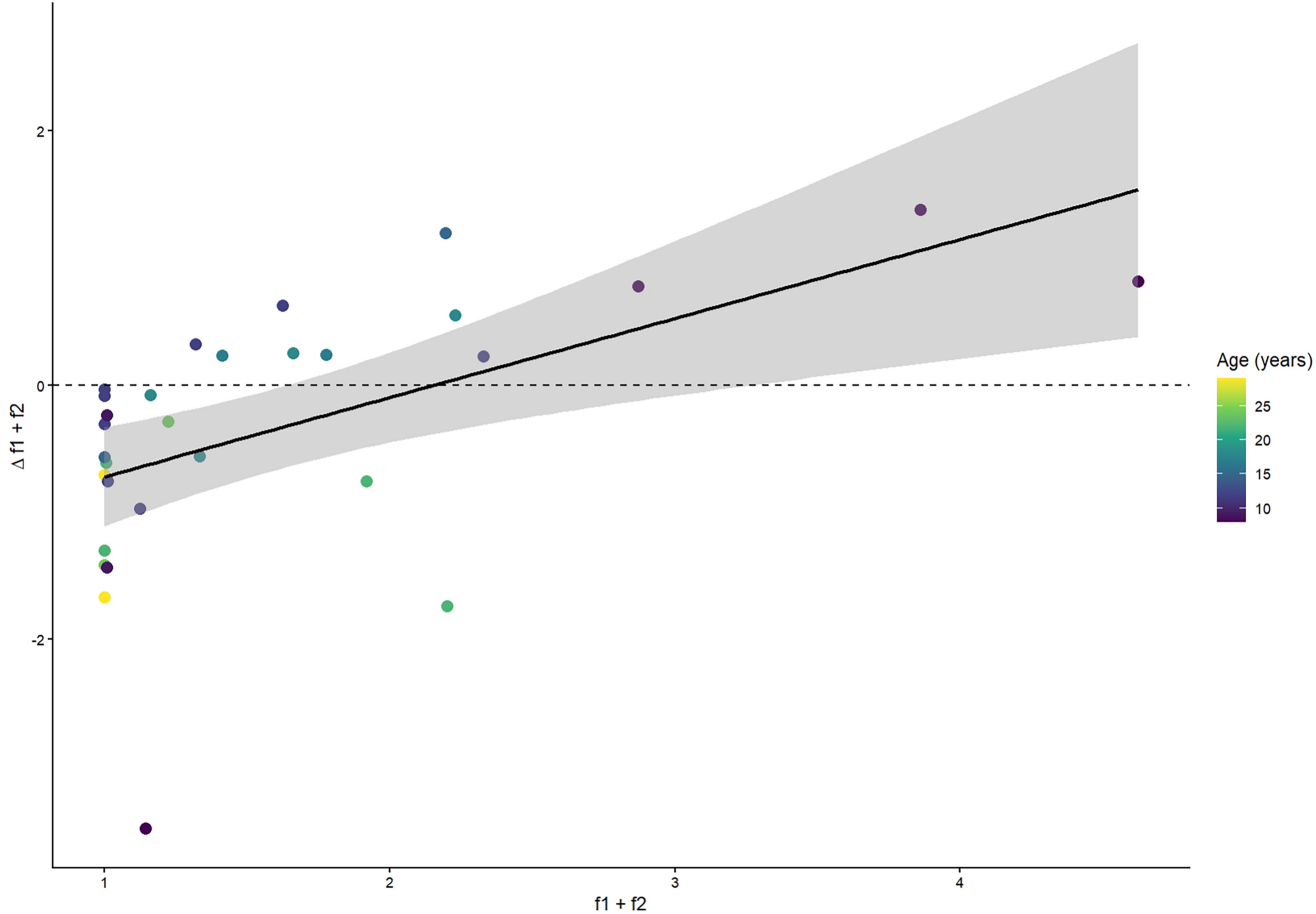
Correlation between baseline f1+f2 amplitude and Δf1+f2. Each colored dot represents an individual participant. The positive correlation suggests that baseline binocular interaction strength predicts the magnitude of short-term monocular deprivation (STMD)-induced plasticity.

Baseline f*1*+f*2* amplitude was positively correlated with the magnitude of post-STMD change (Figure 7), suggesting its predictive value.

Age was not significantly correlated with the change in f*1*+f*2* amplitude following either amblyopic-eye STMD (*r* = −0.01, *p* = 0.98) or fellow-eye STMD (*r* = −0.60, *p* = 0.18), suggesting that within the age range studied (8–25 years), the observed plasticity effects were not primarily driven by age.

## 4 Discussion

Short-term monocular deprivation (STMD) has been shown to rapidly alter binocular plasticity in adults with amblyopia, likely through shifts in the excitatory/inhibitory balance within the visual cortex, leading to disinhibition and altered interocular contrast gain control. Our findings demonstrate the efficacy of STMD in modulating both monocular and, more importantly, binocular SSVEP responses in the amblyopic eye. This underscores the role of STMD in triggering visual plasticity in amblyopia. Notably, we observed a slight discrepancy with a previous study regarding the fellow eye effect on the second day of STMD (Hu et al., 2023). This divergence may be attributed to differences in visual stimuli. While a black-and-white checkerboard primarily activates the luminance contrast pathway, the color checkerboard used in our study additionally and potently stimulates the chromatic antagonism pathway. These distinct types of information are processed separately and integrated at different stages of the visual pathway, potentially leading to varied plasticity outcomes as measured in V1 and higher cortical areas.

This study lacks direct behavioral measures of interocular suppression (e.g., contrast-gain control paradigms or perceptual reports during binocular rivalry) to corroborate the SSVEP findings. Therefore, the observed increase in intermodulation SSVEPs should be interpreted as a neural correlate consistent with, but not direct proof of, reduced interocular suppression. Furthermore, the absence of a significant binocular SSVEP change after fellow-eye STMD warrants further investigation. It may indicate that the dominant fellow eye’s influence is less susceptible to short-term perturbation in the amblyopic visual system, or that our SSVEP paradigm is less sensitive to subtle changes induced by STMD of the fellow eye. Second, the STMD order was not counterbalanced. Although the 24-h washout interval exceeds the typical recovery window of STMD effects and baseline measures were stable across days, we cannot completely exclude the possibility of order effects. Future studies employing counterbalanced or crossover designs with extended washout periods (e.g., 48–72 h) are warranted to fully rule out this potential confound. Third, accommodation was not explicitly clamped; however, the fixed viewing distance (1.0 m), use of shutter goggles, and full optical correction minimize its potential impact.

In conclusion, our work objectively elucidates the primary mechanism of STMD-induced visual plasticity in adolescent and adult amblyopes within our sample (aged 8–25 years) using visual electrophysiology. A key finding is that the baseline amplitude at the f_1_+f_2_ frequency correlates with the degree of binocular SSVEP change following STMD, suggesting its potential as a predictive biomarker for treatment response. These results enhance our understanding of the underlying visual cognitive mechanisms. Although the plasticity effects of STMD in adolescent and adults may differ from those during the critical period in children, our findings offer novel perspectives for developing supplementary amblyopia therapies. For instance, STMD-induced short-term ocular dominance plasticity could be combined with existing treatments—such as perceptual learning, electrical stimulation, or magnetic stimulation—to improve overall visual function (both visual acuity and binocularity) in amblyopia. This approach may be particularly beneficial for patients who do not respond adequately to conventional patching therapy. Overall, our results support the use of reverse STMD as a mechanism to reduce interocular suppression in amblyopia. The predictive value of baseline SSVEP measures warrants further investigation for personalizing treatment strategies. Future studies should focus on combining STMD with perceptual learning paradigms to evaluate long-term functional improvements.

## Data Availability

The raw data supporting the conclusions of this article will be made available by the authors, without undue reservation.
